# The roles of motivation, anxiety and learning strategies in online Chinese learning among Thai learners of Chinese as a foreign language

**DOI:** 10.3389/fpsyg.2022.962492

**Published:** 2022-08-16

**Authors:** Wei Xu, Haiwei Zhang, Paisan Sukjairungwattana, Tianmiao Wang

**Affiliations:** ^1^Faculty of Humanities and Social Sciences, City University of Macau, Macao, Macao SAR, China; ^2^School of Chinese as a Second Language, Peking University, Beijing, China; ^3^Faculty of Liberal Arts, Mahidol University, Nakhon Pathom, Thailand

**Keywords:** online language learning, foreign language anxiety, learning motivation, learning strategies, Chinese as a foreign language

## Abstract

The impact of motivation, anxiety and learning strategies on the achievement of foreign language proficiency has been widely acknowledged in the context of traditional offline classroom settings. However, this issue has not been extensively documented in relation to online learning, which has become the predominant form of language learning during the period of the COVID-19 pandemic. The current study was conducted to investigate the relative prediction of motivation, anxiety and learning strategies for second language achievement among 90 Thai adult learners of Chinese as a foreign language (CFL) who took online Chinese courses. The participants completed a questionnaire dealing with motivation, anxiety, learning strategies, and their Chinese proficiency was measured by self-report and a Chinese vocabulary size test. A series of hierarchical regression analyses revealed two major findings. First, anxiety emerged as the most stable factor for the participants' CFL achievement, followed by learning strategies and motivation. Second, motivation, anxiety and learning strategies only significantly predicted the participants' self-rated Chinese language proficiency, but not their performance on the Chinese vocabulary size test. The overall results indicate the relative importance of motivation, anxiety and learning strategies to Chinese language learning in the online environment and suggest different measures of CFL achievement may lead to different research findings. The general findings were of theoretical and pedagogical significance for understanding and addressing individual differences factors in online language learning.

## Introduction

An increase in online education has been accelerated by the COVID-19 pandemic. Traditionally, online learning was viewed as a complement to classroom instruction, but it has become increasingly common across the broad spectrum of education during the COVID-19 period, including second/foreign language learning at universities, and this trend is likely to continue in the near future (Klimova, 2021; Maican and Cocoradă, 2021). Compared with other languages, the demand for the learning of the Chinese language is in full swing globally due to Chinese economic and social importance (Zhong et al., 2021), given that China has maintained steady economic growth during this period. There may thus be a positive outlook regarding the increase of Chinese learners around the world, which calls for greater attention to research concerning Chinese learning (Ma et al., 2017; Gong et al., 2018, 2019).

**Figure 1:**
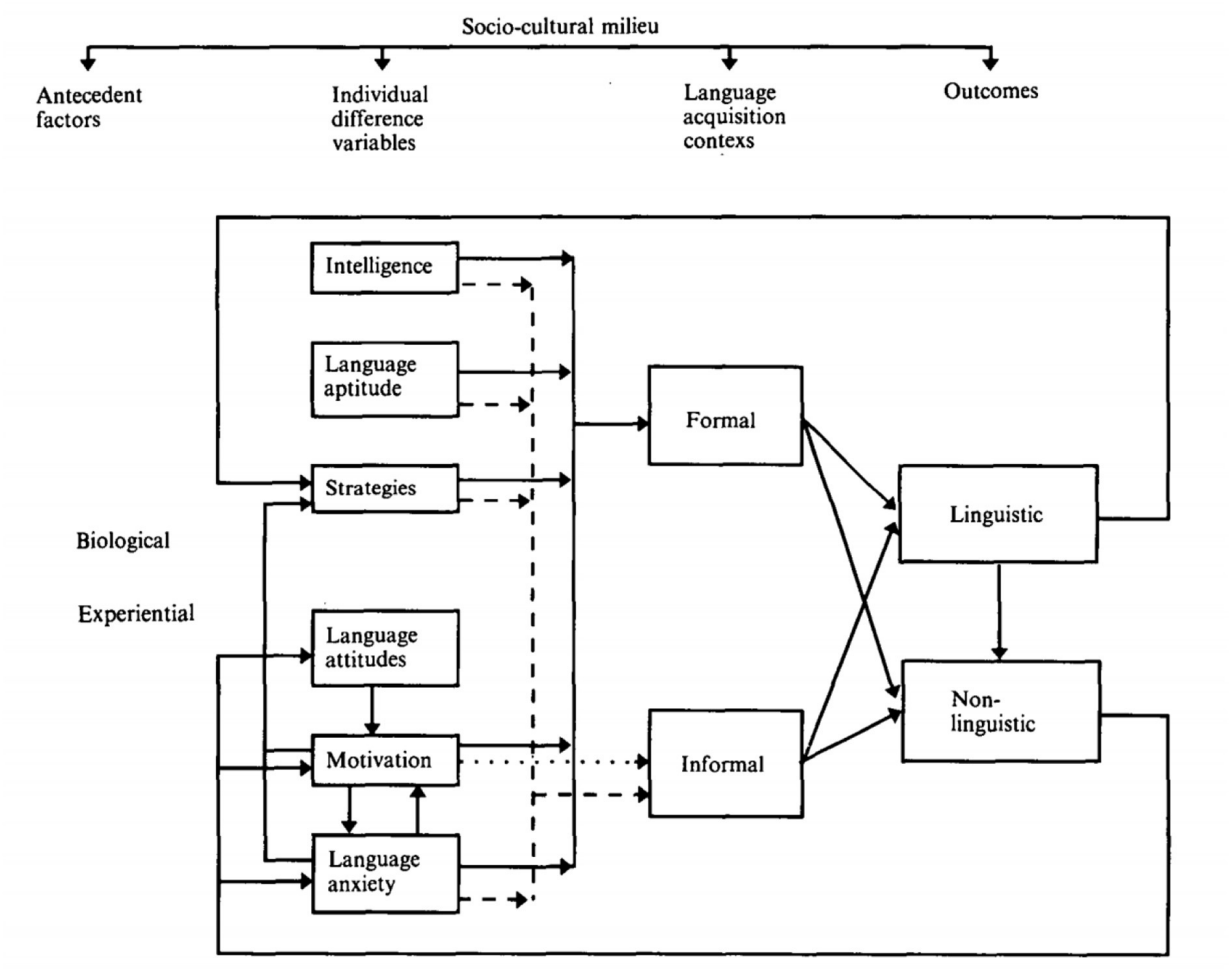
Schematic representation of the Socio-educational Model of SLA (Gardner and MacIntyre, 1993b, p. 8).

The Socio-educational Model of second language acquisition (SLA), proposed by Gardner and MacIntyre (1993b) (Figure 1), suggests that second language (L2) learning outcomes are influenced by antecedent factors, individual differences variables and language acquisition context. Individual differences have been assumed to account for a great deal of the learners' L2 attainment variance and are found to be the most consistent predictors of L2 learning success (Dörnyei, 2005). According to Gardner and MacIntyre (1993b), individual difference variables include intelligence, language aptitude, learning strategies, attitudes, motivation and anxiety. It is important to note that measuring intelligence and language aptitude may encounter unique difficulties in accessing suitable measurement tools and pose some ethical problems (Reed and Stansfield, 2004), and that attitudes and motivation have been integrated with tools such as the Attitude/Motivation Test Battery (Gardner and Smyihe, 1981), therefore, motivation, anxiety and learning strategies could be considered as the three fundamental components of individual differences variables in SLA. In recent studies examining individual differences variables in SLA, motivation, anxiety, and learning strategies were ranked as the top three variables (Lei and Liu, 2018; Zhang, 2019b).

Furthermore, online learners may encounter new challenges in relation to motivation, anxiety and learning strategies. In comparison to the offline classroom settings, for example, online learners may feel less motivated and more anxious due to the lack of intermediate feedback and help from instructors or interaction with their classmates, therefore they are required to be more self-regulated (Ma, 2022; Zahradnikova, 2022; Zhang, 2022). Moreover, these variables (motivation, anxiety and learning strategies) might work differently in the online learning environment (e.g., Estrella, 2022; Mihai et al., 2022). Considering the significant gap between studies on individual differences variables in the two learning contexts (offline vs. online) (Table 1) and the unique features of online learning (Hampel and Stickler, 2015; Russell and Murphy, 2021), as well as the popularity of online learning during the period of COVID-19 pandemic, it is imperative to investigate whether these three individual difference variables affect the achievement of online language learning.

**Table 1:** Results of keyword search on *Web of Science* and *Scopus*.

	**Web of science**		**Scopus**	
**Keywords**	**2018–2022**	**2020–2022**	**2018–2022**	**2020–2022**
Foreign language learning motivation	1,199	421	991	592
Online foreign language learning motivation	115	59	93	67
Foreign language learning anxiety	427	173	348	215
Online foreign language learning anxiety	50	32	34	32
Foreign language learning strategies	1,119	638	2,298	590
Online foreign language learning strategies	122	50	97	72

The data indicates the numbers of articles that have been searched and was accessed on 16th March, 2022.

## Literature review

As is known, each of the constructs of motivation, anxiety and learning strategies could be decomposed into different components, which might exert different influences on SLA (Dörnyei, 2005). As a result of the limited space and the focus of the present study, the review section concentrated on the influence of the general constructs of motivation, anxiety and learning strategies, rather than the sub-dimensions of each construct, on L2 learning. Furthermore, according to the model proposed by Gardner and MacIntyre (1993b) (Figure 1), motivation influences anxiety, which in turn influences learning strategies. Thus, following such a path, this section reviewed studies about motivation, anxiety and learning strategies, respectively.

### Motivation in foreign language learning and CFL learning

Motivation to learn a new language could be defined from a distinctly behavioral perspective as the effort individuals exert to learn due to a “desire” to and in seeking “satisfaction” from the experience (Gardner, 1985). Many researchers have explored how motivation works using various theoretical frameworks (Boo et al., 2015), including the instrumental and integrative orientations (Gardner, 1985), intrinsic and extrinsic motivations (Noels et al., 2000) and the ideal and ought-to L2 selves (Papi, 2010; Papi and Teimouri, 2012, 2014), and it has been widely acknowledged that motivated learners tend to outperform the less motivated ones in SLA (Dörnyei, 2005; Papi, 2018; de Burgh-Hirabe, 2019; Gong et al., 2020; Sudina, 2021).

In their meta-analysis of 75 studies, Masgoret and Gardner (2003) found that most studies showed a moderate correlation coefficient (*r* = 0.39) between motivation and L2 achievement, and this relationship did not differ significantly across the learning contexts (second vs. foreign language) and ages of the participants. However, it remains unclear whether this relationship applies to online education. As an example, Ushida (2005b) found that learners' motivation ratings were significantly correlated with their module test scores and their performance in online chat sessions at the end of the semester among L2 learners of French and Spanish. In contrast, Lin et al. (2017) observed that high-school students' final grades in an online course were not predicted by intrinsic or extrinsic motivation. These different findings may be attributable to factors such as the research contexts, recruited participants and the timing of the study, indicating that more research is necessary to investigate the role of motivation in online learning in different contexts.

Research on motivation to learn Chinese language has mainly concentrated on topics such as the construct of Chinese as a second language (CSL) or CFL learning motivation (Cheng, 1993; Wen, 1997, 2011; Comanaru and Noels, 2009), the influencing factors of CSL/CFL learning motivation (Lu and Li, 2008; Chua et al., 2009; Cai and Zhu, 2012; Ruan et al., 2015; Ji et al., 2017; Gong et al., 2020), and the comparison between Chinese-as-heritage-language (CHL) and non-CHL learners' motivation in North America (Lu and Li, 2008; Comanaru and Noels, 2009; Wen, 2011). A limited number of studies have examined the relationship between motivation and Chinese language proficiency, but with inconsistent results. For instance, among the CFL learners in the U.S., Wen (1997) found that intrinsic motivation was a significant predictor of Chinese course achievement (*n* = 77), and Lu and Li (2008) reported a similar finding concerning integrative motivation and test scores (*n* = 120). However, it appears that research about the CFL learners in less developed countries produces different results from American CFL learners. As an example, Ter et al. (2020) reported a negative relationship between integrative motivation and self-rated Chinese competence and a positive relationship between instrumental motivation and self-rated Chinese competence among 205 undergraduate CFL learners in Malaysia, and Zheng and Richard (2021) observed a weak relationship between motivation and Chinese academic achievement among 164 Grade 6 students in Thailand.

### Anxiety in foreign language learning and CFL learning

Anxiety is a critical aspect of effect in language learning and plays an imperative role in SLA (Horwitz, 2016, 2017; Oxford, 2017; Gregersen, 2020; Sudina, 2021; Jiang and Papi, 2022; Pan et al., 2022). In general, foreign language anxiety (FLA) is defined as “a distinct complex construct of self-perceptions, beliefs, feelings, and behaviors related to classroom language learning arising from the uniqueness of language learning process” (Horwitz et al., 1986, p. 128). It has been proposed that FLA exerts a negative influence on language learning performance through a variety of mechanisms, including Krashen's affective filter hypothesis (Krashen, 1981, 1982), the three analogies of FLA (communication apprehension, fear of negative evaluation, and test anxiety) put forward by Horwitz (2016, 2017), MacIntyre and Gardners' model of causality between anxiety and L2 learning (1989) and the dynamic nature of anxiety (Gregersen, 2020). Irrespective of these different theoretical frameworks, the negative influence of FLA on L2 learning performance has been widely documented (Botes et al., 2020; Russell, 2020; Dikmen, 2021; Sudina, 2021). A recent meta-analysis article based on 69 studies from 14 countries found that FLA showed a relatively strong yet negative correlation coefficient with EFL performance (*r* = −0.61), and that this relationship did not vary significantly across the grade level or country (Dikmen, 2021). However, most of the previous studies were conducted in the traditional offline classrooms, and less attention has been paid to FLA in the context of online learning (Russell, 2020). In other studies, the components of online FLA (Wang and Zhang, 2021) and the resources for online FLA (Coryell and Clark, 2009) have been evaluated, as well as the comparison of FLA between classroom and distance learning (Pichette, 2009), but no studies have investigated whether the negative association between FLA and L2 learning performance holds true in an online context.

Gardner and MacIntyre (1993a) further found the impact of different measurements of L2 achievement on research findings related to this issue. Gardner and MacIntyre examined the associations between anxiety and various measures of language achievement in French, and reported a higher negative correlation coefficient between anxiety and self-rated proficiency than that between anxiety and objective measures such as cloze test and word production. Gardner and MacIntyre pointed out that measures of anxiety were linked to learners' concerns about their perceived inadequacy, as evidenced in the close relationship between low-levels of anxiety and different types of positive emotions such as self-confidence (Clément, 1986), hope, optimism and agency (Oxford, 2017), which would probably have a greater influence on subjective self-ratings than on objective tests. Thus, different measures were recommended by Gardner and MacIntyre to assess learners' L2 achievement in exploring the role anxiety plays in SLA.

A variety of approaches have been taken to explore Chinese learning anxiety. In terms of participants, researchers have examined the issue of Chinese learning anxiety among CHL learners (Xiao and Wong, 2014; Luo, 2015) and CFL learners (Luo, 2014a,b; Zhou, 2017; Sung and Li, 2019) in the U.S. and Thailand (Zheng and Richard, 2021), and CSL learners in China (Basith et al., 2019). Based on the scope of learning anxiety, some studies focused on Chinese learning anxiety (Luo, 2013, 2015; Basith et al., 2019; Sung and Li, 2019; Zheng and Richard, 2021), some focused on a specific language skill, such as reading anxiety (Zhao et al., 2013; Zhou, 2017), writing anxiety (Xiao and Wong, 2014) and speaking anxiety (Luo, 2014b). According to most studies, anxiety was negatively associated with Chinese language achievement across a variety of contexts, although several studies did not report such a significant relationship (Zhao et al., 2013; Zheng and Richard, 2021). Learners who have been learning Chinese for longer times and have higher proficiency levels tend to experience lower levels of anxiety, which has been consistently found among CSL learners in China (Basith et al., 2019), CHL learners (Luo, 2015) and CFL learners (Luo, 2012, 2014a,b; Zhao et al., 2013; Zhou, 2017) in the U.S.

### Learning strategies in foreign language learning and CFL learning

The importance of language learning strategies for SLA has been widely acknowledged (e.g., Chu et al., 2015; Galti, 2016; Chou, 2018; Ngo, 2019; Gao, 2020; Gong et al., 2021a). Several articles have systematically reviewed research on language learning strategies since 2010 (Oxford et al., 2014; Rose et al., 2018; Plonsky, 2019; Zhang et al., 2019; Teng and Zhang, 2021). Previous research has explored language learning strategies from a variety of perspectives, including listening comprehension (e.g., Ross and Rost, 1991; Thompson and Rubin, 1996; Carrier, 2003; Santos et al., 2008; Ngo, 2019), oral communication or speaking (e.g., O'Malley and Chamot, 1990; Sun et al., 2016; Chou, 2018), reading comprehension (e.g., Kern, 1989; Anderson, 1991), vocabulary (Alemi and Tayebi, 2011; Gu, 2019), and writing (e.g., Yang and Plakans, 2012; De Silva, 2015). In general, both offline and online learning contexts found these different learning strategies to be useful and helpful for achieving learning objectives. In addition to the various theoretical frameworks pertaining to learning strategies, self-regulation has been gaining attention (Oxford, 2016; Rose et al., 2018; Teng and Zhang, 2021) and has been applied to online learning. Self-regulated learning has been commonly defined as the learners' efforts to regulate their learning process in order to achieve specific learning goals (Zimmerman and Schunk, 2001; Oxford, 2016). As an example, Barnard-Brak et al. (2010) developed the Online Self-Regulated Learning Questionnaire and observed that learners' academic achievement differed significantly depending on their learning strategies. An et al. (2021) further found that self-regulated strategy significantly predicted ESL learners' learning outcomes and enjoyment of English.

Chinese language learners have also been studied in relation to their learning strategies (Jiang and Cohen, 2012; Gong et al., 2021b). Some studies focused on learning strategies for Chinese characters (Liu and Jiang, 2003; Shen, 2005; Sung and Wu, 2011), vocabulary (Tam and Kim, 2021), speaking (Sun et al., 2016), communication (Wang et al., 2021a) and cultural adaption to study-abroad (Gong et al., 2020, 2021c). Furthermore, the relationship between strategy use and Chinese language performance has been well-established in previous studies. For instance, learners' metacognitive strategies, such as self-regulation through monitoring their progress, preserving tasks and setting realistic goals, were positively associated with learners' Chinese academic achievement among CFL learners in the U.K. (Wang et al., 2009), and compensation strategy was more commonly used in higher proficiency group than in less successful CSL learners in Taiwan area (Chu et al., 2015). In addition, this relationship has also been found in young CFL learners in Spain, whose affective strategies showed the strongest correlation with their Youth Chinese Test scores (Cáceres-Lorenzo, 2015). Similar findings were also observed in research concerning Chinese characters (Shen, 2005) and speaking (Sun et al., 2016). However, whether these findings hold true in the online learning context is still unclear.

### Combining motivation, anxiety and learning strategies in L2 learning

A limited number of studies examined the relative prediction of motivation, anxiety and learning strategies for SLA among English language learners, and reported similar findings to some extent (Brown et al., 2001; Hou, 2017). Brown et al. (2001) studied the relationships among five variables (personality, motivation, anxiety, learning strategies, and language proficiency) and found that motivation was one of the most reliable predictors for distinguishing between the low and middle/high proficiency groups among ESL learners. Similarly, Hou (2017) reported that EFL learners' English proficiency was only significantly linked with their motivation, as opposed to their strategy or anxiety. However, studies among CFL learners in the context of online learning have identified some different conclusions (Lin et al., 2017; Zheng and Richard, 2021). Lin et al. (2017) found, for instance, that online learning strategies operated at a moderate level in the process of foreign language learning and predicted the learners' perceived progress and final grades, but motivation was not a significant predictor of Chinese language learning performance. The study by Zheng and Richard (2021) revealed a weak relationship between motivation, anxiety and Chinese academic achievement among young Thai CFL learners. These different findings suggest the necessity of conducting more studies in different learning contexts.

### Research on online CFL learning

With the development of computer technology in the 1970's, computer-assisted Chinese language learning emerged, following along the same development path as applications of computer-assisted language learning in other foreign languages education (Da and Zheng, 2018; Zhang, 2019a; Zhou, 2020). As a result of the COVID-19 pandemic, CFL researchers have made considerable efforts to explore the challenges and opportunities brought about by online Chinese learning. Some leading Chinese journals such as *Chinese Teaching in the World* (世界汉语教学, Shijie hanyu jiaoxue) and *Language Teaching and Linguistics Studies* (语言教学与研究, Yuyan jiaoxue yu yanjiu) have organized several forums focusing on the strategies for overcoming the challenges of online CFL learning and research (Li et al., 2020; Lu et al., 2020a,b; Ba et al., 2021).

CFL researchers from different countries outside China carried out various case studies about remote Chinese teaching (Zhang, 2021a; Liu, 2022). Several researchers further conducted empirical studies about online CFL learning. For instance, Qing and Diamantidaki (2020) explored the CFL learners' learning experience in the UK from the perspectives of cognitive presence, social presence and teaching presence and found that the online Mandarin courses were highly valued by the learners. Based on Positive Psychology, Wang and Jiang (2022) found that CFL learners showed a high level of foreign language enjoyment (FLE) in the online learning context, yet they did not find a significant relationship between FLE and the participants' Chinese language achievement, as measured by a 10-point self-report scale and an objective Chinese language test. Despite the fact that both studies explored learners' experience of online Chinese learning, they focused on a specific aspect (such as language test method or learning enjoyment), while neglecting the learners' motivation, anxiety and learning strategies, which are important to provide us with a clearer picture of the CFL online learning.

The context of online learning may present new challenges and difficulties for CSL/CFL learners compared to the traditional offline classroom (Gao, 2020). Online students, for example, were more likely to experience difficulties concentrating and to feel more stressed and anxious than offline students (Ba et al., 2021; Ma, 2022; Zahradnikova, 2022). Regarding motivation, studies have found a significant prediction of motivation in CFL learners' learning performance, such as online learning self-efficacy and learning progress (Ushida, 2005a; Hong et al., 2017). Despite some studies finding limited effects of online learning settings on the change of motivation (Cai and Zhu, 2012), many researchers have clearly observed that learners' motivation in the context of online learning might decrease or even disappear due to the reduced outside-class interaction, therefore maintaining self-motivation is particularly crucial for learners' success in online learning (Li et al., 2020; Lin, 2022; Ma, 2022; Zahradnikova, 2022; Zhang, 2022). In terms of learning strategies, CFL learners might need to develop new strategies for learning Chinese in the context of online learning, such as constant self-testing and self-regulated learning strategies (Qian et al., 2018; Lu et al., 2020a; Zhang, 2022). In sum, the online learning context might influence CFL learners' profile in their individual differences factors, thus a study that explores various factors is needed.

### CFL research in less developed countries

In parallel with the rapid growth of CFL learners around the world, the number of studies pertaining to CFL learning has also been on the rise (Ma et al., 2017; Gong et al., 2018, 2019). In 2020, the number of CFL learners was expected to reach 25 million.^1^ Nevertheless, the existing studies have focused mainly on Chinese language learners in mainland China and given relatively little attention to Chinese language learners in other countries (Gong et al., 2018), specifically in less developed countries. For instance, only two of the 14 chapters in a recently edited book entitled *Teaching the Chinese Language Remotely: Global Cases and Perspectives* (Liu, 2022), addressed less developed countries such as South Africa and Mauritius, while the other 10 chapters focused on China and developed countries. As far as the authors are aware, CFL learners from less developed countries are overlooked by researchers for a variety of reasons, such as the lack of CFL researchers or professional CFL research networks including journals and researcher associations.

Thailand has the largest number of Chinese language learners among less developed countries, which has been estimated to have exceeded 1 million in 2021 (Fu, 2021), owing to its close economic, cultural and political ties with China. Like the global trend during the COVID-19 pandemic, Chinese language learning has shifted to online platforms in Thailand. Thailand, however, lags behind other countries in the development of information and communication technologies,^2^ which may pose particular difficulties to online language learning, which in turn may affect language learning performance. However, relevant research on the impact of individual differences factors on CFL learning in Thailand is lacking (Zheng and Richard, 2021). Therefore, investigating how Thai CFL learners learn Chinese online could have both theoretical and practical implications for online Chinese language education.

Finally, as discussed above in this section, some gaps remain in the exploration of motivation, anxiety and learning strategies for L2 learning performance in the online context. First of all, most previous research has concentrated on a particular aspect of motivation, anxiety and learning strategies, and there has not been a comprehensive study that integrates these factors. Secondly, most of the existing studies focused on English language learners and paid less attention to learners of other languages, such as Chinese learning in less developed countries. Therefore, an investigation into the role of motivation, anxiety and learning strategies in the online settings among CFL learners in the less developed countries outside China is necessary.

## The current study

To fill these gaps, the current study aimed to examine the role of motivation, anxiety and learning strategies in L2 Chinese achievement among Thai CFL learners. The results of such a study could provide more empirical evidence for research on the role of individual differences factors in SLA in the online context, and could provide pedagogical implications for successful Chinese teaching and learning in the online settings in less developed countries. Specifically, the current study seeks to answer the following questions:

RQ1. How does language learning motivation predict CFL learning performance?
RQ2. How does foreign language anxiety predict CFL learning performance?
RQ3. How do learning strategies predict CFL learning performance?
RQ4. In what ways are motivation, anxiety and learning strategies different in the prediction of CFL learning performance?

## Method

### Participants

The participants were 90 local undergraduates of different grades majoring in Chinese language (Mean age = 19.21, SD = 1.19; 11 males and 79 females) from three universities in Thailand (Table 2). The average length of CFL learning (measured from the onset of their CFL learning to November 2021) was 3.76 years (SD = 3.19). Due to the interruption in sitting HSK tests during the COVID-19 pandemic, the participants were not able to report their Chinese language proficiency based on HSK scores. Self-assessment can be a good indicator in foreign language learning (Li et al., 2006). Therefore, they were required to self-assess their Chinese language proficiency on the basis of a 7-point Likert scale, with 1 representing elementary level, 4 for intermediate level and 7 for advanced level. As seen in Table 2, the results of the one-sample *t*-tests indicated that the participants' self-rated Chinese language proficiency and its four sub-skills were significantly below intermediate level (midscale 4), suggesting that the participants generally perceived their Chinese language proficiency as falling in between elementary and intermediate levels.

**Table 2:** Summary of the participants' self-rated Chinese language proficiency.

	**Min**	**Max**	**Mean**	**SD**	**Mode**	**Median**	**Skewness**	**Kurtosis**	***t*** **-test results**
Speaking	1	7	3.27	1.15	3	3	0.43	0.17	*t*(89) = 6, *p < 0.0*01, Cohen's d = 0.63
Listening	1	7	3.58	1.23	4	4	−0.05	−0.26	*t*(89) = 3.24, *p* = 0.002, Cohen's d = 0.34
Reading	1	7	3.52	1.13	3	3	0.18	0.16	*t*(89) = 4, *p < 0.0*01, Cohen's d = 0.42
Writing	1	7	2.99	1.16	3	3	0.41	0.59	*t*(89) = 8.23, *p < 0.0*01, Cohen's d = 0.87
Overall proficiency	1	7	3.47	1.15	4	4	0.04	0.19	*t*(89) = 4.39, *p < 0.0*01, Cohen's d = 0.46

It is noteworthy that there was an imbalance between the percentage of males and females, and this may be due to the fact that females generally outnumbered their male counterparts when learning CFL, which may be common in other foreign languages as well. However, the impact of gender on age [*t*(14.3) = 0.67, *p* = 0.51, Cohen's d = 0.20], length of CFL learning [*t*(12) = 1.05, *p* = 0.31, Cohen's d = 0.31] and self-rated Chinese language proficiency [*t*(11.2) = 0.99, *p* = 0.34, Cohen's d = 0.36] was not significant and the effect sizes of gender were small (Cohen, 1988; Plonsky and Oswald, 2014), suggesting that the male and female participants were homogeneous in CFL learning experience and Chinese language proficiency.

### Instruments

Four instruments were designed to collect the data on motivation, anxiety, learning strategies, and Chinese learning achievement among the CFL learners.

#### Motivation

Thai CFL learners' learning motivation was tested employing a widely used questionnaire with 18 items (Cronbach's α = 0.77) revised from Noels et al. (2000), which included two categories of motivation: intrinsic motivation (IM) and extrinsic motivation (EM). IM was divided into three categories, including knowledge, accomplishment, and stimulation; EM included external regulation, introjected regulation, and identified regulation. An example item was “I study Chinese for the pleasure that I experience in knowing more about the literature of the second language group” (see Appendix 1).

#### Anxiety

Learners' online learning anxiety was assessed by Online Chinese Learning Anxiety Scale (OCLAS, Cronbach's α = 0.79). The four-scaled questionnaire was revised from Luo (2015) and was designed to assess the learners' anxiety about Chinese language speaking, listening, reading, and writing during their online study. In the questionnaire, there were 16 items divided into four subscales. An example item was “During my online class, I feel very self-conscious about speaking Chinese in front of other students” (see Appendix 2).

#### Learning strategies

Participants' online learning strategies were assessed using a 24-item questionnaire (Cronbach's α = 0.84) revised from Barnard-Brak et al. (2010). It focused on six aspects: goal setting, environment structuring, task strategies, time management, help seeking, and self-evaluation. An example item was “I set standards for my assignments in online courses” (see Appendix 3).

The questionnaires on motivation, anxiety and learning strategies required the participants to respond on a 5-point Likert scale ranging from “1-strongly disagree” to “5-strongly agree.” A double check was performed on the ratings of the questionnaires, and any response that was in doubt was reviewed by two authors.

#### Chinese learning achievement

It is no doubt that learners' L2 proficiency could be best assessed using a comprehensive standardized test, such as HSK (Hanyu shuiping kaoshi, Chinese proficiency test) for Chinese. However, these tests are difficult to obtain and are time-consuming to administer, making them ineffective for research purposes (Zhang et al., 2020b). More importantly, the outbreak of COVID-19 pandemic has interrupted the administration of standardized language tests. In addition, the recruited participants came from different universities, thus relying on their course grades was not appropriate. Considering that a variety of measures of L2 proficiency could lead to different research findings (Gardner and MacIntyre, 1993a; Zhang et al., 2020b), in keeping with previous studies (Gardner and MacIntyre, 1993a; Wang and Jiang, 2022), the participants' Chinese learning achievement was assessed both objectively and subjectively.

Regarding the objective measurement, the participants' vocabulary size was used as an indicator of their Chinese language achievement for several reasons. First, the vocabulary size test has repeatedly been found to have a strong association with overall L2 proficiency (Nation and Anthony, 2017; Miralpeix and Muñoz, 2018) and specific sub-skills (Stæhr, 2008), thus being utilized as a reliable and valid independent measure of L2 proficiency assessment (Park et al., 2022) or a placement indicator (Zhang et al., 2020a). Second, CSL/CFL researchers have not reached a consensus on the optimal method for assessing Chinese language proficiency (Zhang, 2018, 2021b; Zhang et al., 2020b). Finally, it is not possible to offer standardized proficiency or placement tests or cloze tests to CFL learners in the context of online learning. As a result, an online vocabulary size test was developed and employed, considering that it is simple to design, administer and grade in terms of research purposes. A total of 127 items were included in the online Chinese vocabulary size test. These items were systematically selected from the *Chinese Proficiency Grading Standards for International Chinese Language Education* (2021), an official syllabus published by the Center for Language Education and Cooperation in China. The target items in the test were well-balanced in terms of frequency, difficulty and word class. The participants were required to translate the displayed Chinese words into Thai. One point was assigned to a correct response and zero points to an incorrect response or an unanswered question. The raters were two Thai CSL learners who were graduate students in a Chinese university and passed the highest level of the HSK test. The inter-rater reliability was 0.95. The accuracy rate was calculated by dividing the number of accurate responses by 127. The Cronbach alpha reliability of this test was 0.90.

Considering a subjective measure of L2 achievement, as seen in the popular application of the Language History Questionnaire (Li et al., 2006) and Language Experience and Proficiency Questionnaire (Kaushanskaya et al., 2019) in research concerning SLA, self-assessment may be considered to be a reliable indicator of L2 proficiency level in both English (Brown and Hudson, 1998; Hulstijn, 2012; Park et al., 2022) and Chinese language learners (Zhang et al., 2020b). Consequently, in the present study, participants were asked to self-rate their overall Chinese language proficiency and their proficiency in subskills (e.g., listening, speaking, reading and writing) on a seven-point Likert scale, where one indicates an elementary level and seven indicates an advanced level.

### Administration

The data were collected from August to September 2021, and the participants learned Chinese completely online due to the influence of the COVID-19 pandemic in Thailand since the beginning of 2020. Instructions in higher education have been redirected to online platforms as a result of the pandemic. The online courses were administered synchronically with formal instructions. The participants were asked to complete an online questionnaire about their motivation, anxiety and learning strategies for learning Chinese online, the 58 items of which were randomly ordered. The questionnaire was presented in Thai. The questionnaire was piloted among five CFL learners before it was formally administered in order to ensure that the items were understandable. The five participants in the pilot study were from Thai universities and represented a range of Chinese language proficiency levels (2 beginners, 2 intermediates, and 1 advanced). Upon completing the questionnaire, the participants were required to complete an online Chinese vocabulary size test. They took an average of 31.07 min to complete both the questionnaire and the vocabulary size test. All the participants were informed and approved of the use of the collected data for this research, and were debriefed that the questionnaires would not affect their assessments or tests and that they could withdraw from the research at any time when necessary.

## Results

### Descriptive results

In Table 3, the scores of the participants in motivation, anxiety, learning strategies and vocabulary size test are presented. The participants displayed above-midscale (3) ratings in anxiety, intrinsic motivation, extrinsic motivation and learning strategies.

**Table 3:** Scores in motivation, anxiety, learning strategies and vocabulary size test.

**Measured variable**	**Min**	**Max**	**Mean**	**SD**	**Mode**	**Skewness**	**Kurtosis**
Anxiety	2.06	5	3.35	0.71	3.19	0.05	−0.05
Intrinsic motivation	3	5	4.21	0.60	4	−0.44	−0.49
Extrinsic motivation	2.78	5	3.67	0.62	3.44	0.41	0.03
Learning strategies	2.71	5	3.65	0.44	3.46	0.12	0.10
Accuracy rate in vocabulary size test	0.02	0.77	0.28	0.20	0.08	0.43	−0.98

### Results of regression analysis

A structural equation modeling (SEM) analysis would be the most appropriate method to address the four research questions. However, the data did not enable a model with good fit indicators, perhaps as a result of the relatively small sample size (*n* = 90) in the present study. Therefore, a series of hierarchical regression analyses were conducted to answer RQ1-4. In the first series of regression analyses, the dependent variable was self-rated Chinese language proficiency. In the 1^st^ step, the participants' background variables including age, gender, ethnicity, grade and length of CFL learning were added to the base model as independent variables. The 2^nd^, 3^rd^ and 4^th^ steps involved entering the participants' ratings of motivation, anxiety and learning strategies in different orders into the model. In the second series of regression analyses, the dependent variable was the accuracy rate of the Chinese vocabulary size test and the independent variables were added in the same way as in the first series of regression analyses. The correlation matrix between the measured variables is displayed in Table 4. The results of the first series of regression analyses are presented in Tables 5–10 (see Appendices), and the results of the second series of regression analyses are displayed in Tables 11–16 (see Appendices).

**Table 4:** Correlation matrix between the measured variables.

	**1**	**2**	**3**	**4**	**5**	**6**	**7**	**8**	**9**	**10**	**11**
Self-rated Chinese proficiency	—										
Vocabulary size test accuracy rate	0.51^***^	—									
Gender	0.14	0.13	—								
Age	0.02	−0.06	−0.01	—							
Ethnic Chinese background	0.27^*^	0.17	0.12	−0.01	—						
Grade	0.04	−0.08	−0.06	0.72^***^	−0.13	—					
Length of CFL learning	0.53^***^	0.40^***^	0.13	0.06	0.11	0.02	—				
Anxiety	−0.24^*^	−0.10	−0.12	−0.17	−0.14	−0.12	−0.06	—			
Intrinsic motivation	0.25^*^	−0.08	−0.09	0.16	−0.01	0.16	0.06	−0.05	—		
Extrinsic motivation	0.10	0.04	−0.05	−0.18	−0.01	−0.06	0.08	0.32^**^	0.51^***^	—	
Learning strategies	0.17	−0.01	−0.12	0.03	−0.02	0.06	−0.05	0.24^*^	0.51^***^	0.53^***^	—

* p < 0.05,

** p < 0.01,

and

*** p < 0.001.

The regression analysis utilized the participants' average scores in anxiety, intrinsic motivation, extrinsic motivation and learning strategies, rather than the scores in each subsection. This is due to the fact that there were 90 participants in the present study, which means that the maximum number of independent variables could be nine, based on a recommended ratio of 10:1 between sample size and the number of independent variables used in conducting regression analysis (Maxwell, 2000; Knofczynski and Mundfrom, 2007). In addition, both intrinsic and extrinsic motivation were entered separately because the two types of motivation could lie on different ends of a continuum and exert influences on L2 learning in different ways (Noels et al., 2000).

In regard to RQ1 about the prediction of motivation, it accounted for 5% of the variance (Δ*F* = *3.21, p* = 0.05; β_IM_ = 0.27, *p* = 0.02; β_EM_ = −0.09, *p* = 0.40) of self-rated Chinese language proficiency when controlling the participants' background variables (i.e., gender, age, ethnic background, grade and the length of CFL learning) (Model 2 vs. Model 1 in Tables 5, 6). Motivation did not significantly predict the accuracy rates of the Chinese vocabulary size test (Tables 11–16).

As for RQ2 about the prediction of anxiety, it significantly accounted for 5% of the variance of self-rated Chinese language proficiency (β = −0.22, *p* = 0.02; Δ*F* = 5.54*, p* = 0.02) when controlling the participants' background variables (Model 2 vs. Model 1 in Tables 7, 8), 4% of the variance (β = −0.21, *p* = 0.04; Δ*F* = 4.44*, p* = 0.02) after controlling the participants' background variables and motivation (Model 3a vs. Model 2 in Tables 5, 6), 7% of the variance (β = −0.28, *p* = 0.003; Δ*F* = *9.16, p* < 0.01) when the participants' background variables and learning strategies were controlled for (Model 3a vs. Model 2 in Tables 9, 10), and 4% of the variance (β = −0.24, *p* = 0.02; Δ*F* = 5.88*, p* = 0.02) after controlling the participants' background variables, motivation and learning strategies (Model 4b vs. Model 3b in Tables 5–10). However, anxiety was not a significant predictor of accuracy rates of the Chinese vocabulary size test (Tables 11–16).

With respect to RQ3 about the prediction of learning strategies, they accounted for 4% of the variance of self-rated Chinese proficiency (β = 0.20, *p* = 0.04; Δ*F* = 4.37*, p* = 0.04) when controlling the participants' background variables (Model 2 vs. Model 1 in Tables 9, 10), and 6% of the variance (β = 0.26, *p* = 0.01; Δ*F* = 7.95*, p* = 0.01) when the participants' background variables and anxiety were controlled for (Model 3b vs. Model 2 in Tables 7, 8). On the Chinese vocabulary size test, participants' learning strategies did not significantly predict their performance (Tables 11–16).

RQ4 relates to the relative predictive power of motivation, anxiety, and learning strategies on achievement in L2 Chinese. In terms of self-rated Chinese language proficiency, the full model (Model 4b in Tables 5, 7, 9) which includes the three measured variables as well as the background variables significantly accounted for 45% of the variance (adjusted *R*^2^ = 0.38), *F*(9, 72) = 6.55, *p* < 0.001. Based on the three measured variables, anxiety significantly predicted self-rated Chinese language proficiency (β=.-24, *p* = 0.02, *R*^2^ = 0.06), the significant prediction of learning strategies achieved a marginal level (β = 0.22, *p* = 0.06, *R*^2^ = 0.03), but neither intrinsic motivation (β = 0.14, *p* = 0.24, *R*^2^ = 0.03) nor extrinsic motivation (β = −0.06, *p* = 0.60, *R*^2^ = 0.01) was a significant predictor. The regression equation of the model was

*Self-rated Chinese language proficiency*=*0.09gender -0.13age* +*0.43ethnic background*+*0.01grade*+*0.18length of CFL learning*+*0.32intrinsic motivation -0.17extrinsic motivation -0.49anxiety* +*0.56 learning strategies*

As for the accuracy rates of Chinese vocabulary size test, the full model (Model 4b in Tables 11, 13, 15) significantly explained 24% of the variance (adjusted *R*^2^ = 0.14), *F*(9, 72) = 2.52, *p* = 0.01. However, none of the three measured variables significantly predicted the accuracy rates of Chinese vocabulary size test: anxiety, β = −0.17, *p* = 0.16, *R*^2^ = 0.02; learning strategies, β = 0.13, *p* = 0.32, *R*^2^ = 0.005; intrinsic motivation, β = −0.21, *p* = 0.13, *R*^2^ = 0.01; extrinsic motivation, β = 0.14, *p* = 0.32, *R*^2^ = 0.01. The regression equation of the model was

*Accuracy rates of Chinese vocabulary size test*=*0.06gender -0.01age* +*0.04ethnic background-0.02grade*+*0.02length of CFL learning-0.09intrinsic motivation* +*0.07extrinsic motivation -0.06anxiety* +*0.06 learning strategies*

As seen above, each of the three measured variables explained 4% to 7% of the variance, which represents small effect sizes (Cohen, 1988). It was evident that anxiety was the most stable predictor, followed by learning strategies and motivation.

In terms of the participants' background variables, the length of Chinese language learning was consistently significant for predicting both self-rated Chinese language proficiency (β = 0.50~0.52, *p* < 0.001) and the accuracy rates of the Chinese vocabulary size test (β = 0.36~0.38, *p* < 0.001). In the full models (Model 4b in Tables 5–16), the length of Chinese language learning accounted for the largest percentage of variance in self-rated Chinese language proficiency (*R*^2^ = 0.26) and the accuracy rates of Chinese vocabulary size test (*R*^2^ = 0.15) among all the independent variables.

## Discussion

The present study explored the prediction of motivation, anxiety and learning strategies for learning achievement among Thai CFL learners in the context of online learning. The results of the hierarchical regression analysis revealed that, regarding the participants' self-rated Chinese language proficiency, anxiety (Model 2 in Table 7, Model 3a in Tables 5, 10, Model 4a and Model 4b in Tables 5–10) was the most stable yet negative predictor among the three individual differences variables, whereas learning strategies (Model 2 in Table 9, Model 3b in Table 7) and motivation (Model 2 in Table 5) were to some extent positive predictors. In spite of this, none of the three variables significantly predicted the participants' accuracy rates in the Chinese vocabulary size test. Furthermore, the significant prediction of the length of Chinese language learning in the two measures of L2 Chinese achievement was stable across different models, which is reasonable considering that a longer period of language learning generally means greater input and output in various components of language and sub-skills.

### Anxiety and CFL learning

In the current study, participants showed moderate anxiety in the context of online learning, and their anxiety had a negative correlation with and prediction for self-rated Chinese language proficiency, which is generally consistent with previous studies involving language learners of English (MacIntyre and Gardner, 1991; Horwitz, 2001; Dikmen, 2021), Japanese (Aida, 1994), Spanish (Coryell and Clark, 2009) and Chinese (Luo, 2013; Zhao et al., 2013; Luo, 2014a,b, 2015; Zhou, 2017; Basith et al., 2019; Zheng and Richard, 2021). Although some early research observed a positive influence of anxiety on L2 learning (Chastain, 1975; Kleinmann, 1977), and researchers have not reached a general consensus about the underlying mechanism for the negative impact of anxiety on SLA (Krashen, 1981, 1982; Horwitz et al., 1986; MacIntyre and Gardner, 1989, 1994; Gregersen, 2020), the negative impact of foreign language anxiety on L2 achievement has been widely recognized, such as reduction in cognitive performance (Gregersen, 2020), self-confidence (Chou, 2018), willingness to communicate (Basith et al., 2019), and this negative influence could be observed at different stages (e.g., input, processing and output) of second language learning (MacIntyre and Gardner, 1994).

CFL proficiency, and this correlation coefficient was as high as−0.61 among learners of EFL in several less developed countries (Dikmen, 2021). Similarly, Tanielian (2014) reported a weak and negative correlation (*r* = −0.16) between classroom anxiety and English performance in a study on Thai EFL learners. It is possible that the low correlation between anxiety and foreign language achievement in Thailand is due to cultural values within the country. There is a greater emphasis placed on fun and pleasure among Thai people than on achievement and ambition (Komin, 1991). The words *achievement* and *ambition* even have negative connotations in Thai language (Punyapiroje and Morrison, 2007). The country of Thailand is also a Buddhist country, and Buddhism has been found to positively influence happiness (Gray, 2012; Senasu and Singhapakdi, 2017). In 2020, Thailand ranked second in Southeast Asia according to the Global Happiness Levels report. The influence of anxiety on foreign language achievement might be diminished by a higher level of subjective happiness and a lower level of ambition/achievement orientation. Nonetheless, this explanation should be considered tentative and should be supported by additional empirical evidence in the future.

Compared to previous research, the present study found a relatively smaller effect size of anxiety on language achievement (*r* = −0.21). A noteworthy finding of the present study is that in comparison with the research on anxiety of CFL learners in the offline learning context in the U.S. (Luo, 2013), the Thai learners showed a relatively higher level of anxiety (M = 3.35). There may be two possible reasons for this. First, the present study was conducted during the COVID-19 pandemic, which may have increased the participants' anxiety about learning. A comparative study found that Thai university students showed the highest levels of anxiety during the pandemic, compared with participants from Indonesia and Taiwan (Pramukti et al., 2020). Second, it is possible that a higher level of anxiety is caused by the online learning environment. In contrast to anxiety experienced in traditional offline classrooms (Luo, 2012; Kasbi and Elahi Shirvan, 2017), anxiety in the context of online foreign language learning could be caused by factors related to teachers, learners, technology and family environment (Ushida, 2005a; Coryell and Clark, 2009; Pichette, 2009; Hampel and Stickler, 2015; Adedoyin and Soykan, 2020; Russell and Murphy, 2021). For instance, online learners may not have immediate interaction with instructors and peers when feedback or help is needed. In the context of online learning at home during the COVID-19 pandemic, those lacking motivation or self-discipline might become distracted by stimuli (e.g., video games, Internet surfing, etc.) not related to learning at home, which makes it challenging to keep up with learning. In addition, some online learners with low socioeconomic status may not have access to resources, such as Internet infrastructure, mobile data, electronic devices and isolated study spaces. All of these factors may lead to the emergence and persistence of anxiety in online language learning, which may further add emotional pressure and negatively influence the learners' learning performance.

### Learning strategies and CFL learning

Using the strategy questionnaire, it was found that online Thai CFL learners were aware of their learning and could utilize various strategies actively to aid their CFL learning, as indicated by their above-midscale scores. Although learning strategies did not significantly correlate with L2 Chinese achievement (see the correlation matrix in Table 4), a significant prediction of learning strategies for self-rated Chinese language proficiency was observed after controlling for the participants' background variables and anxiety. These findings are consistent with those reported by learners of English (Ngo, 2019; An et al., 2021) and Chinese in the offline contexts (Shen, 2005; Wang et al., 2009; Cáceres-Lorenzo, 2015; Chu et al., 2015; Sun et al., 2016), as well as in the online learning context (Lin et al., 2017). It has been widely acknowledged that self-regulated learning strategies are multifaceted and could exert their influence on learning achievements *via* cognitive, metacognitive, behavioral and self-motivational channels (Zimmerman and Schunk, 2001). The presence of COVID-19 requires learners to be more self-regulatory at home than in an offline setting. This is due to the unique characteristics of online learning such as a lack of supervision and lack of immediate peer interaction (Lin, 2022). This account aligns with the findings reported by Holcomb et al. (2004) who argued for self-regulation as a critical component of distance education success.

### Motivation and CFL learning

As discussed in earlier sections, the participants were compelled to enroll in online learning under the influence of the COVID-19 pandemic, rather than of their own will, thus whether the participants' motivation is indicative of their actual level of inner psychological wellbeing could be questioned. Prior studies have shown that learners' language learning motivation can remain stable across offline and online settings, suggesting a limited effect of the learning context on the change of motivation (Cai and Zhu, 2012). This implies that the measurement tools used in the present study could tap into the participants' online Chinese learning motivation to some extent.

Participants in the current study showed that they had a strong intrinsic motivation and moderate extrinsic motivation to learn Chinese and that their intrinsic motivation levels significantly correlated with and further significantly predicted their self-rated Chinese language proficiency after controlling for their background variables. However, this significant predictive power of motivation disappeared after further controlling for anxiety and learning strategies in the regression model. In addition, motivation ratings did not significantly predict the participants' performances on the vocabulary size test. These findings are consistent with previous research that reported a weak or even null relationship between motivation and Chinese language learning among CFL learners in Thailand (Zheng and Richard, 2021) and the U.S. (Lin et al., 2017), however, they are inconsistent with findings reported among ESL learners (Brown et al., 2001; Hou, 2017). These four studies were conducted among learners in non-target-language contexts, such as Chinese language learners in Thailand (Zheng and Richard, 2021) the U.S. (Lin et al., 2017) and the English learners in Japan (Brown et al., 2001) and Taiwan (Hou, 2017). Thus, these inconsistent findings indicate the necessity of conducting further studies to explore this issue in the future.

According to the present study, intrinsic motivation is a stronger predictor of language proficiency than extrinsic motivation, which is consistent with findings reported in previous research (Noels et al., 2000; Noels, 2001; Hong et al., 2017; Sun and Gao, 2020). Intrinsic motivation “generally refers to motivation to engage in an activity because that activity is enjoyable and satisfying to do” (Noels et al., 2000, p. 61) and builds upon innate needs for competence and self-determination (Deci and Ryan, 1985). By contrast, extrinsic motivation closely relates to the motive to achieve some instrumental objectives. Online learning occurs during the COVID-19 pandemic without immediate supervision from instructors or peer interaction, making self-regulation and self-determination more important than in offline settings (Holcomb et al., 2004; Lin, 2022). The significant prediction of intrinsic motivation in self-rated Chinese language proficiency aligns with the significant influence of self-regulated learning strategies, suggesting the importance of self-management for the performance in online learning.

Motivation plays a lesser role in second language achievement than anxiety or learning strategies, which may be explained by the following reasons. Firstly, as discussed in the section on anxiety and language learning, Thai cultural characteristics may be a contributing factor, such as a tendency to prefer fun and pleasure over achievement and ambition (Komin, 1991; Punyapiroje and Morrison, 2007). There may be some reduction in the contribution of motivation to language learning achievement as a result of this. The second reason may relate to the indirect influence of motivation on language learning. As Gardner and MacIntyre (1993b) and Gardner et al. (1997) proposed, motivation was assumed to play an indirect role in second language achievement *via* anxiety and strategy. To be more specific, motivation to learn a foreign language might be an initial psychological trait. Increased motivation may lead the learners to explore appropriate learning strategies and build stronger self-confidence, which in turn alleviate language learning anxiety and facilitate the development of second language abilities (Chu, 2008; Nishitani and Matsuda, 2011; Zarei, 2014; Galti, 2016; Hou, 2017). However, this account is tentative, studies with a larger sample size might offer more conclusive results.

### Different measures of CFL learning achievement and different research results

The overall findings of the present study are consistent with those reported in previous research (Gardner and MacIntyre, 1993a; Zhang, 2018; Zhang et al., 2020b; Park et al., 2022). Among the participants in the present study, motivation, anxiety and learning strategies significantly predicted their self-rated Chinese language proficiency to some extent, yet none of these variables significantly predicted their performance on the Chinese vocabulary size test. Observations of different roles of anxiety across the two measures of Chinese language learning achievement were consistent with those described by Gardner and MacIntyre (1993a), who found a higher negative correlation coefficient between anxiety and self-rated proficiency than that between anxiety and objective measures such as cloze tests and word production. These findings are also in agreement with those of Zhang (2018) and Zhang et al. (2020b), who noted that a comprehensive measurement of second language achievement would be more informative than a measure focusing on a single aspect, such as reading. It may be possible to explain the discrepancy between the two measures from the following perspectives.

First, based on the measurement approach taken in this study, motivation, anxiety, and learning strategies were not specific to vocabulary learning, so their effects may not be evident in a specific area of Chinese language achievement such as receptive vocabulary size. In general, vocabulary learning strategies focus on beliefs about vocabulary learning, inference, the use of dictionaries, taking notes, rehearsal, encoding, and active use (Gu, 2019); however, this study focused on general online learning strategies.

Second, Thai CFL learners recruited were at elementary and intermediate levels, and they possessed a limited vocabulary size, as evidenced by their relatively low accuracy rate on the vocabulary size test (mean = 0.28). Additionally, the participants studied Chinese for different lengths of time, which may have contributed to the variability in the accuracy rate of the vocabulary size test (SD = 0.20).

Third, there is also the possibility of the Dunning-Kruger effect (Dunning, 2011; Trofimovich et al., 2014; Saito et al., 2020), a phenomenon in which unskilled performers tend to overestimate their abilities. Further analysis revealed that the number of participants who scored in the first quartile in the vocabulary test and self-rated as 3, 4 and 5 was 10, 4 and 1, while the number of participants who scored in the second quartile in the vocabulary test and self-rated as 3, 4 and 5 was 9, 6 and 1. There was a clear overestimation of Chinese language proficiency among some participants with lower vocabulary size. There is a possibility that the Dunning-Kruger effect may have an impact on research findings concerning the role of individual differences factors in second language acquisition.

### Theoretical and pedagogical implications

The overall findings of the present study provide theoretical implications for understanding the role of motivation, anxiety and learning strategies in SLA in the context of online learning. The general results partially support the importance of individual differences factors (such as anxiety and learning strategies) in L2 achievement in the Socio-educational Model of L2 learning (Gardner and MacIntyre, 1993b), as identified among CFL learners in their context of online learning. In addition, the overall findings suggest the different predictive powers of motivation, anxiety and learning strategies in second language achievement.

In terms of anxiety and learning strategies, the present study provides substantial evidence of the significance of these two individual differences factors in SLA from the perspective of CFL learners. The overall findings of the present study suggest that anxiety and learning strategies might override motivation, which could predict SLA indirectly through other variables such as learning strategies or anxiety. The results of anxiety and motivation suggest the importance of considering cultural and situational background in researching language learning anxiety across different countries (Horwitz, 1999). The smaller effect size of anxiety and the lower contribution of motivation to CFL achievement may be due to the fun/pleasure-oriented cultural values in Thailand. Although it is widely acknowledged that most of the language learning theories originate from developed countries, whether these theories could be applied successfully in less developed countries is still unclear and more supporting evidence from an ecological perspective across different cultures and contexts is required (Larsen-Freeman, 2018).

The present study has practical implications for the instruction of Chinese language in less developed countries. A first recommendation is that instructors pay more attention to anxiety and learning strategies than to motivation. It was found that anxiety and learning strategies contributed more to CFL achievement than motivation. Therefore, instructional strategies could be tailored to ease learners' anxiety about CFL learning and train them to develop self-regulating strategies for learning a foreign language online. A supportive and positive instructional approach can reduce learners' language learning anxiety (Young, 1991; Vogely, 1998; Oxford, 2017; Jin et al., 2021). If instructors are working in less developed countries, they should consider the local culture when taking targeted measures to address anxiety. As an example, instructors in Thailand may utilize Buddhism tenets to ease learners' anxiety related to language learning. According to an instructor of the CFL learners in the present study, there was no formal instruction in language learning strategies provided to them. Learners are therefore encouraged to become familiar with the benefits of self-regulated learning strategies and to increase their online learning autonomy from the perspectives of motivation, affect, cognition, and social interaction (Oxford, 2016). Additionally, instructors in less developed countries should take into account the relatively poor internet infrastructure in order to address issues related to anxiety and learning strategies, and traditional communication strategies such as telephone or mail may be suitable for facilitating learner-instructor interactions.

A second recommendation is to use multiple measures of language proficiency when assessing learners. There may be a Dunning-Kruger effect in self-rated language proficiency based on the results of the study of the influence of different measurements on the research results. It is therefore necessary to pay more attention to the practice of reporting second language proficiency (Zhang, 2018; Zhang et al., 2020b; Park et al., 2022). As a result of these findings, it is imperative that both subjective as well as objective measures of achievement be included in studies of second languages (Gardner and MacIntyre, 1993a; Trofimovich et al., 2014). In order to measure Chinese language proficiency objectively, researchers may use the HSK test, the cloze test, the character recognition test, or the vocabulary size test (Zhang, 2018, 2021a; Zhang et al., 2020b, 2021, 2022).

## Conclusions and limitations

The present study explored the predictions of motivation, anxiety and learning strategies for Chinese language achievement among Thai CFL learners during the COVID-19 pandemic. The current study was conducted during the COVID-19 pandemic and it was one of few attempts to examine motivation, anxiety, learning strategies and learning achievement from the perspective of online Chinese learning. The general results of the present study are consistent with the findings reported in previous studies, which furthered our understanding of the relationships between L2 learning achievement and individual differences factors in the contexts of offline and online language learning. Nevertheless, some limitations of the current study cannot be ignored.

The first limitation concerns the questionnaire and measurement. The questionnaire and the vocabulary size test in the present study consisted of 58 and 127 items, respectively. These lengthy tasks, as well as the online data collection method, may result in some loss of accuracy (Gosling et al., 2004; Chetverikov and Upravitelev, 2016). Due to a lack of face-to-face interaction between the teacher and the learner, some participants may have used online dictionaries in the vocabulary test or did not take the questionnaire seriously. Further, there was a limitation in the questionnaire for measuring motivation and learning strategies, as they did not examine the two constructs specific to the online context or Chinese language. Also, the vocabulary size test and self-rated Chinese language proficiency may not reflect the participants' real achievement in online language learning, which could be determined using grade scores or teachers' ratings alternatively. Additionally, the present study employed a softer approach limited by the online learning environment during the pandemic to rate the participants' responses to the vocabulary size test, and this practice might affect the research results (Webb, 2008).

The second limitation concerns the participants. Five participants were piloted and a total of 90 participants were recruited due to various difficulties encountered during the period of the COVID-19 pandemic. As the majority of participants were below the intermediate level, the results may not be generalized to those at the advanced level. In addition, a larger sample size will be desirable in future studies to explore the roles of different sub-dimensions of motivation, anxiety and learning strategies in SLA. Moreover, a large sample including participants from different countries or cultural backgrounds would facilitate the analysis of the differences and similarities in anxiety, motivation, and learning strategies across different contexts.

Thirdly, while the present study focused on three individual differences factors, it is known that online learning achievement could be influenced by a wide range of factors related to students (e.g., information literacy), teachers (e.g., instructional strategies), online platform (e.g., technical convenience and user-friendliness). Consequently, future studies could explore the interaction effect of these different variables on L2 achievement to depict a clearer picture of online learning, or explore from the perspective of positive psychology, whose significance for foreign language learning has been widely documented (e.g., Mercer and MacIntyre, 2014; Dewaele et al., 2019; Wang, 2021; Wang et al., 2021b; Baatouche et al., 2022). To triangulate the results in the future, it is suggested that a mixed method of research that includes both quantitative and qualitative data such as interviews or open-ended comments be used (Riazi and Candlin, 2014; Mackey and Bryfonski, 2018).

## Data availability statement

The original contributions presented in the study are included in the article/Supplementary material, further inquiries can be directed to the corresponding author/s.

## Ethics statement

Ethical review and approval was not required for the study on human participants in accordance with the local legislation and institutional requirements. Written informed consent from the participants' legal guardian/next of kin was not required to participate in this study in accordance with the national legislation and the institutional requirements.

## Author contributions

WX: conceptualization, methodology, questionnaire, data analysis, draft writing, and editing. HZ: conceptualization, methodology, questionnaire, data analysis, draft writing, reviewing, and editing. PS: conceptualization, investigation, questionnaire, and data analysis. TW: methodology, reviewing, and editing. All authors contributed to the article and approved the submitted version.

## Funding

This study was funded by the Center for Language Education and Cooperation in 2021 (21YH04A) and the Ministry of Education of China (20YJC740088).

## Conflict of interest

The authors declare that the research was conducted in the absence of any commercial or financial relationships that could be construed as a potential conflict of interest.

## Publisher's note

All claims expressed in this article are solely those of the authors and do not necessarily represent those of their affiliated organizations, or those of the publisher, the editors and the reviewers. Any product that may be evaluated in this article, or claim that may be made by its manufacturer, is not guaranteed or endorsed by the publisher.

## Footnotes

^1^ https://news.cgtn.com/news/2020-12-16/Over-70-countries-incorporate-Chinese-into-national-education-systems-WgFixEeAMw/index.html

^2^https://www.huawei.com/minisite/gci/en/country-profile-th.html; https://tcdata360.worldbank.org/indicators/h2f85e6e7?indicator=24721andviz=bar_chartandyears=2016
