# Supplementary material for: The roles of motivation, anxiety and learning strategies in online Chinese learning among Thai learners of Chinese as a foreign language
Source: Front Psychol. 2022 Aug 16;13:962492. doi: 10.3389/fpsyg.2022.962492 (PMC9426342; doi:10.3389/fpsyg.2022.962492)
Supplement: Supplementary file 1 [file Data_Sheet_1.pdf]

Appendices-Tables

Table 5 Summary of hierarchical regression analysis (dependent variable: self-rated Chinese language proficiency; motivation entered as the 2nd step)

Model	Variable	B	SE	p	β	B	SE	p	β	B	SE	p	β	B	SE	p	β	B	SE	p	β	B	SE	p	β
1	Gender	.02	.37	.97	01	.14	.37	.71	.04	.01	.36	.97	.00	.09	.36	.81	.02	.21	.37	.57	.05	.09	.36	.81	.02
	Age	-.08	.13	.54	-.08	-.12	.13	.35	-.13	-.11	.13	.40	-.12	-.13	.13	.29	-.14	-.15	.13	.27	-.15	-.13	.13	.29	-.14
	Ethnic	.52	.23	.03	.45	.51	.23	.03	.21	.44	.23	.06	.18	.43	.22	.05	.18	.51	.23	.03	.21	.43	.22	.05	.18
	Grade	.05	.21	.81	.03	.03	.20	.87	.02	-.01	.20	.96	-.01	.01	.19	.94	.01	.06	.20	.78	.04	.01	.19	.94	.01
	Length	.18	.03	< .001	.51	.17	.03	< .001	.51	.17	.03	< .001	.50	.18	.03	< .001	.52	.18	.03	< .001	.52	.18	.03	< .001	.52
2	IM					.63	.25	.02	.27	.48	.26	.07	.21	.32	.27	.24	.14	.51	.26	.05	.22	.32	.27	.24	.14
	EM					-.25	.29	.40	-.09	.02	.31	.95	.01	-.17	.32	.60	-.06	-.43	.31	.18	-.16	-.17	.32	.60	-.06
3a	ANX									-.43	.20	.04	-.21	-.49	.20	.02	-.24								
4a	STR													.56	.29	.06	.22								
3b	STR																	.45	.30	.13	.17	.56	.29	.06	.22
4b	ANX																					-.49	.20	.02	-.24
Model		$F(5, 76)=7.60$, $p<.001$				$F(7, 74)=6.66$, $p<.001$				$F(8, 73)=6.65$, $p<.001$				$F(9, 72)=6.55$, $p<.001$				$F(8, 73)=6.22$, $p<.001$				$F(9, 72)=6.55$, $p<.001$			
		$R^2=.33$, Adjusted $R^2=.29$				$R^2=.39$, Adjusted $R^2=.33$				$R^2=.42$, Adjusted $R^2=.36$				$R^2=.45$, Adjusted $R^2=.38$				$R^2=.41$, Adjusted $R^2=.34$				$R^2=.45$, Adjusted $R^2=.38$			

Note. ethnic: ethnic Chinese background; length: length of CS/FL learning; IM: intrinsic motivation; EM: extrinsic motivation; STR: strategies.

Table 6 Summary of model comparisons (dependent variable: self-rated Chinese language proficiency; motivation entered as the 2nd step)

Model	Model	ΔR²	F	df1	df2	p
1	2	0.05	3.21	2	74	0.05
2	3a	0.04	4.44	1	73	0.04
3a	4a	0.03	3.74	1	72	0.06
2	3b	0.02	2.31	1	73	0.13
3b	4b	0.04	5.88	1	72	0.02

Table 7 Summary of hierarchical regression analysis (dependent variable: self-rated Chinese language proficiency; anxiety entered as the 2nd step)

Model	Variable	B	SE	p	β	B	SE	p	β	B	SE	p	β	B	SE	p	β	B	SE	p	β	B	SE	p	β
1	Gender	.02	.37	.97	01	-.14	.37	.7	-.12	.01	.36	.97	0	.09	.36	.81	.02	.05	.36	.90	.01	.09	.36	.81	.02
	Age	-.08	.13	.54	-.08	-.1	.13	.42	-.11	-.11	.13	.4	-.12	-.13	.13	.29	-.14	-.11	.12	.36	-.12	-.13	.13	.29	-.14
	Ethnic	.52	.23	.03	.45	.45	.23	.06	.39	.44	.23	.06	.18	.43	.22	.05	.18	.42	.22	.06	.17	.43	.22	.05	.18
	Grade	.05	.21	.81	.03	.02	.2	.9	.02	-.01	.2	.96	-.01	.01	.19	.94	.01	.01	.19	.95	.01	.01	.19	.94	.01
	Length	.18	.03	< .001	.51	.18	.03	< .001	.51	.17	.03	< .001	.5	.18	.03	< .001	.52	.18	.03	< .001	.52	.18	.03	< .001	.52
2	Anxiety					-.45	.19	.02	-.22	-.43	.2	.04	-.21	-.49	.2	.02	-.24	-.57	.19	.00	-.28	-.49	.20	.02	-.24
3a	IM									.48	.26	.07	.21	.32	.27	.24	.14								
	EM									.02	.31	.95	.01	-.17	.32	.6	-.06								
4a	STR													.56	.29	.06	.22								
3b	STR																	.67	.24	.01	.26	.56	.29	.06	.22
4b	IM																					.32	.27	.24	.14
	EM																					-.17	.32	.60	-.06
Model		$F(5, 76)=7.60$, $p<.001$				$F(6, 75)=7.63$, $p<.001$				$F(8, 73)=6.65$, $p<.001$				$F(9, 72)=6.55$, $p<.001$				$F(7, 74)=8.28$, $p<.001$				$F(9, 72)=6.55$, $p<.001$			
		$R^2=.33$, Adjusted $R^2=.29$				$R^2=.38$, Adjusted $R^2=.33$				$R^2=.42$, Adjusted $R^2=.36$				$R^2=.45$, Adjusted $R^2=.38$				$R^2=.44$, Adjusted $R^2=.39$				$R^2=.45$, Adjusted $R^2=.38$			

Note. ethnic: ethnic Chinese background; length: length of CS/FL learning; IM: intrinsic motivation; EM: extrinsic motivation; STR: strategies.

Table 8 Summary of model comparisons (dependent variable: self-rated Chinese language proficiency; anxiety entered as the 2nd step)

Model	Model	ΔR^2	F	df1	df2	p
1	2	0.05	5.54	1	75	0.02
2	3a	0.04	2.68	2	73	0.08
3a	4a	0.03	3.74	1	72	0.06
2	3b	0.06	7.95	1	74	0.01
3b	4b	0.01	0.71	2	72	0.49

Table 9 Summary of hierarchical regression analysis (dependent variable: self-rated Chinese language proficiency; strategy entered as the 2nd step)

Model	Variable	B	SE	p	β	B	SE	p	β	B	SE	p	β	B	SE	p	β	B	SE	p	β	B	SE	p	β
1	Gender	.02	.37	.97	01	.19	.37	.61	.16	.05	.36	.90	.04	.09	.36	.81	.08	.21	.37	.57	.18	.04	.09	.36	.81
	Age	-.08	.13	.54	-.08	-.08	.13	.51	-.09	-.11	.12	.36	-.12	-.13	.13	.29	-.14	-.15	.13	.27	-.15	-.12	-.13	.13	.29
	Ethnic	.52	.23	.03	.45	.51	.23	.03	.44	.42	.22	.06	.37	.43	.22	.05	.37	.51	.23	.03	.44	.37	.43	.22	.05
	Grade	.05	.21	.81	.03	.05	.20	.81	.03	.01	.19	.95	.01	.01	.19	.94	.01	.06	.20	.78	.04	.01	.01	.19	.94
	Length	.18	.03	< .001	.51	.18	.03	<.001	.52	.18	.03	< .001	.52	.18	.03	< .001	.52	.18	.03	< .001	.52	.18	.03	< .001	.52
2	STR					.51	.24	.04	.20	.67	.24	.006	.26	.56	.29	.06	.22	.45	.30	.13	.17	.56	.29	.06	.22
3a	ANX									-.57	.19	.003	-.28	-.49	.20	.02	-.24								
4a	IM													.32	.27	.24	.14								
	EM													-.17	.32	.60	-.06								
3b	IM																	.51	.26	.05	.22	.32	.27	.24	.14
	EM																	-.43	.31	.18	-.16	-.17	.32	.60	-.06
4b	ANX																					-.49	.20	.02	-.24
Model		F(5, 76)=7.60, p<.001				F(6. 75)=7.34, p<.001				F(7, 74)=8.28, p<.001				F(9, 72)=6.55, p<.001				F(8, 73)=6.22, p<.001				F(9, 72)=6.55, p<.001			
		R^2=.33, Adjusted R^2=.29				R^2=.37, Adjusted R^2=.32				R^2=.44, Adjusted R^2=.38				R^2=.45, Adjusted R^2=.38				R^2=.41, Adjusted R^2=.34				R^2=.45, Adjusted R^2=.38			

Note. ethnic: ethnic Chinese background; length: length of CS/FL learning; IM: intrinsic motivation; EM: extrinsic motivation; STR: strategies.

Table 10 Summary of model comparisons (dependent variable: self-rated Chinese language proficiency; strategy entered as the 2nd step)

Model	Model	ΔR^2	F	df1	df2	p
1	2	0.04	4.37	1	75	0.04
2	3a	0.07	9.16	1	74	0.00
3a	4a	0.01	0.71	2	72	0.49
2	3b	0.04	2.17	2	73	0.12
3b	4b	0.04	5.88	1	72	0.02

Table 11 Summary of hierarchical regression analysis (dependent variable: accuracy rate in vocabulary size test; motivation entered as the 2nd step)

Model	Variable	B	SE	p	β	B	SE	p	β	B	SE	p	β	B	SE	p	β	B	SE	p	β	B	SE	p	β
1	Gender	.07	.07	.34	.34	.07	.07	.36	.33	.05	.07	.49	.25	.06	.07	.43	.29	.08	.07	.32	.37	.06	.07	.43	.29
	Age	-.01	.03	.62	-.07	-.01	.03	.85	-.03	-.003	.03	.90	-.02	-.01	.03	.82	-.04	-.01	.03	.79	-.04	-.01	.03	.82	-.04
	Ethnic	.05	.05	.32	.22	.05	.05	.32	.22	.04	.05	.43	.18	.04	.05	.43	.18	.05	.05	.32	.23	.04	.05	.43	.18
	Grade	-.01	.04	.77	-.05	-.01	.04	.73	-.05	-.02	.04	.63	-.07	-.02	.04	.68	-.06	-.01	.04	.78	-.04	-.02	.04	.68	-.06
	Length	.02	.01	< .001	.38	.02	.01	< .001	.37	.02	.01	< .001	.36	.02	.01	< .001	.38	.02	.01	< .001	.38	.02	.01	<.001	.38
2	IM					-.05	.05	-.97	.34	-.07	.05	.20	-.17	-.09	.06	.13	-.21	-.06	.05	.26	-.15	-.09	.06	.13	-.21
	EM					.05	.06	.91	.37	.09	.06	.18	.19	.07	.07	.32	.14	.03	.06	.59	.07	.07	.07	.32	.14
3a	ANX									-.05	.04	.19	-.15	-.07	.04	.16	-.17								
4a	STR													.06	.06	.32	.13								
3b	STR																	.05	.06	.44	.10	.06	.06	.32	.13
4b	ANX																					-.07	.04	.16	-.17
Model		F(5, 76)=3.76, p=.004				F(7, 74)=2.82, p=.01				F(8, 73)=2.71, p=.01				F(9, 72)=2.52, p=.01				F(8, 73)=2.53, p=.02				F(9, 72)=2.52, p=.01			
		R^2=.20, Adjusted R^2=.15				R^2=.21, Adjusted R^2=.14				R^2=.23, Adjusted R^2=.1				R^2=.24, Adjusted R^2=.14				R^2=.22, Adjusted R^2=.13				R^2=.24, Adjusted R^2=.14			

Note. ethnic: ethnic Chinese background; length: length of CS/FL learning; IM: intrinsic motivation; EM: extrinsic motivation; STR: strategies.

Table 12 Summary of model comparisons (dependent variable: accuracy rate in vocabulary size test; motivation entered as the 2nd step)

Model	Model	ΔR^2	F	df1	df2	p
1	2	0.01	0.58	2	74	0.56
2	3a	0.02	1.73	1	73	0.19
3a	4a	0.01	1.00	1	72	0.32
2	3b	0.01	0.60	1	73	0.44
3b	4b	0.02	2.12	1	72	0.15

Table 13 Summary of hierarchical regression analysis (dependent variable: accuracy rate in vocabulary size test; anxiety entered as the 2nd step)

Model	Variable	B	SE	p	β	B	SE	p	β	B	SE	p	β	B	SE	p	β	B	SE	p	β	B	SE	p	β
1	Gender	.07	.07	.34	.34	.06	.07	.43	.08	.05	.07	.49	.08	.06	.07	.43	.09	.07	.07	.35	.10	.06	.07	.43	.29
	Age	-.01	.03	.62	-.07	-.01	.03	.58	-.08	.00	.03	.90	-.02	-.01	.03	.82	-.04	-.01	.03	.57	-.09	-.01	.03	.82	-.04
	Ethnic	.05	.05	.32	.22	.04	.05	.37	.10	.04	.05	.43	.09	.04	.05	.43	.08	.04	.05	.39	.09	.04	.05	.43	.18
	Grade	-.01	.04	.77	-.05	-.01	.04	.73	-.05	-.02	.04	.63	-.07	-.02	.04	.68	-.06	-.01	.04	.72	-.05	-.02	.04	.68	-.06
	Length	.02	.01	$< .001$	.38	.02	.01	$< .001$	.38	.02	.01	$< .001$	.36	.02	.01	$< .001$	.38	.02	.01	$< .001$	.38	.02	.01	$< .001$	.38
2	Anxiety					-.03	.04	.43	-.09	-.05	.04	.19	-.15	-.06	.04	.15	-.17	-.04	.04	.34	-.11	-.06	.04	.15	-.17
3a	IM									-.07	.05	.20	-.17	-.09	.06	.13	-.21								
	EM									.09	.06	.18	.19	.07	.07	.32	.14								
4a	STR													.06	.06	.32	.13								
3b	STR																	.04	.05	.40	.09	.06	.06	.32	.13
4b	IM																					-.09	.06	.13	-.21
	EM																					.07	.07	.32	.14
Model		$F(5, 76)=3.76$, $p=.004$				$F(6, 75)=3.23$, $p=.007$				$F(8, 73)=2.71$, $p=.01$				$F(9, 72)=2.52$, $p=.01$				$F(7, 74)=2.86$, $p=.01$				$F(9, 72)=2.52$, $p=.01$			
		$R^2=.20$, Adjusted $R^2=.15$				$R^2=.21$, Adjusted $R^2=.14$				$R^2=.23$, Adjusted $R^2=.14$				$R^2=.24$, Adjusted $R^2=.14$				$R^2=.21$, Adjusted $R^2=.14$				$R^2=.24$, Adjusted $R^2=.14$			

Note. ethnic: ethnic Chinese background; length: length of CS/FL learning; IM: intrinsic motivation; EM: extrinsic motivation; STR: strategies.

Table 14 Summary of model comparisons (dependent variable: accuracy rate in vocabulary size test; anxiety entered as the 2nd step)

Model	Model	ΔR^2	F	df1	df2	p
1	2	0.01	0.64	1	75	0.43
2	3a	0.02	1.12	2	73	0.33
3a	4a	0.01	1.00	1	72	0.32
2	3b	0.01	0.72	1	74	0.40
3b	4b	0.03	1.26	2	72	0.29

Table 15 Summary of hierarchical regression analysis (dependent variable: accuracy rate in vocabulary size test; strategy entered as the 2nd step)

Model	Variable	B	SE	p	β	B	SE	p	β	B	SE	p	β	B	SE	p	β	B	SE	p	β	B	SE	p	β
1	Gender	.07	.07	.34	.34	.08	.07	.29	.12	.07	.07	.35	.10	.06	.07	.43	.09	.08	.07	.32	.11	.06	.07	.43	.29
	Age	-.01	.03	.62	-.07	-.01	.03	.62	-.08	-.01	.03	.57	-.09	-.01	.03	.82	-.04	-.01	.03	.79	-.04	-.01	.03	.82	-.04
	Ethnic	.05	.05	.32	.22	.05	.05	.32	.11	.04	.05	.39	.09	.04	.05	.43	.08	.05	.05	.32	.11	.04	.05	.43	.18
	Grade	-.01	.04	.77	-.05	-.01	.04	.76	-.05	-.01	.04	.72	-.05	-.02	.04	.68	-.06	-.01	.04	.78	-.04	-.02	.04	.68	-.06
	Length	.02	.01	< .001	.38	.02	.01	< .001	.38	.02	.01	< .001	.38	.02	.01	< .001	.38	.02	.01	< .001	.38	.02	.01	< .001	.38
2	STR					.03	.05	.52	.07	.04	.05	.40	.09	.06	.06	.32	.13	.05	.06	.44	.10	.06	.06	.32	.13
3a	ANX									-.04	.04	.34	-.11	-.06	.04	.15	-.17								
4a	IM													-.09	.06	.13	-.21								
	EM													.07	.07	.32	.14								
3b	IM																	-.06	.05	.26	-.15	-.09	.06	.13	-.21
	EM																	.03	.06	.59	.07	.07	.07	.32	.14
4b	ANX																					-.06	.04	.15	-.17
Model		F(5, 76)=3.76, p=.004				F(6, 75)=3.18, p=.01				F(7, 74)=2.86, p=.01				F(9, 72)=2.52, p=.01				F(8, 73)=2.53, p=.02				F(9, 72)=2.52, p=.01			
		R^2=.20, Adjusted R^2=.15				R^2=.20, Adjusted R^2=.14				R^2=.21, Adjusted R^2=.14				R^2=.24, Adjusted R^2=.14				R^2=.22, Adjusted R^2=.13				R^2=.24, Adjusted R^2=.14			

Note. ethnic: ethnic Chinese background; length: length of CS/FL learning; IM: intrinsic motivation; EM: extrinsic motivation; STR: strategies.

Table 16 Summary of model comparisons (dependent variable: accuracy rate in vocabulary size test; strategy entered as the 2nd step)

Model	Model	ΔR²	F	df1	df2	p
1	2	0.00	0.42	1	75	0.52
2	3a	0.01	0.94	1	74	0.34
3a	4a	0.03	1.26	2	72	0.29
2	3b	0.01	0.67	2	73	0.52
3b	4b	0.02	2.12	1	72	0.15

Appendix 1: Online Chinese Learning Motivation Scale (OCLMS)
Revised from Noels et al. (2000), originally from Vallerand et al. (1992)

(The questionnaire was presented in Thai, the native language of the participants.)

1 I study Chinese for the pleasure that I experience in knowing more about the literature of the second language group.
2 I study Chinese for the satisfying feeling I get in finding out new things.
3 I study Chinese because I enjoy the feeling of acquiring knowledge about the second language community and their way of life.
4 I study Chinese for the pleasure I experience when surpassing myself in my second language studies.
5 I study Chinese for the enjoyment I experience when I grasp a difficult construct in the second language.
6 I study Chinese for the satisfaction I feel when I am in the process of accomplishing difficult exercises in the second language.
7 I study Chinese for the excitement I feel when hearing foreign languages spoken.
8 I study Chinese for the excitement that I experience while speaking in the second language.
9 I study Chinese for the pleasure I get from hearing the second language spoken by native second language speakers.
10 I study Chinese because I have the impression that it is expected of me.
11 I study Chinese in order to get a more prestigious job later.
12 I study Chinese in order to have a better salary.
13 I study Chinese to show myself that I am a good citizen because I can speak a second language.
14 I study Chinese because I would feel ashamed if I couldn't speak to my friends from the second language community in their native tongue.
15 I study Chinese because I would feel guilty if I didn't know a second language.
16 I study Chinese because I choose to be the kind of person who can speak more than one language.
17 I study Chinese because I think it is good for my personal development.
18 I study Chinese because I choose to be the kind of person who can speak a second language.

Intrinsic Motivations are tested by items 1-9 and 1-3, 4-6, 7-9 indicate factors of *knowledge*, *accomplishment*, and *stimulation* respectively.

Extrinsic Motivations are tested by items 10-18 and 10-12, 13-15, 16-18 indicate factors of *external regulation*, *introjected regulation*, and *identified regulation* respectively.

Appendix 2: Online Chinese Learning Anxiety Scale (OCLAS)
Revised from Luo (2015)

(The questionnaire was presented in Thai, the native language of the participants.)

1	During my online class, it embarrasses me to volunteer answers.
2	During my online class, I can feel my heart pounding when I'm going to be called on.
3	During my online class, I feel very self-conscious about speaking Chinese in front of other students.
4	During my online study, I feel confident when I speak in Chinese.
5	During my online study, I get frustrated when I cannot distinguish among the Chinese tones even after I have worked hard to learn them.
6	During my online study, I get nervous when all the Chinese tones sound the same to me.
7	During my online study, I get anxious when I don't understand what my classmates are saying in Chinese.
8	During my online study, it frightens me when I don't understand what the teacher is saying in Chinese.
9	During my online study, I get so confused when I read Chinese and can't remember what I'm reading.
10	During my online study, I feel confident when I am reading in Chinese.
11	During my online study, I feel intimidated whenever I see a whole page of Chinese in front of me.
12	During my online study, I have difficulty distinguishing among the Chinese characters when reading Chinese.
13	During my online class, I freeze up when I am unexpectedly asked to write Chinese characters.
14	During my online study, writing Chinese characters makes me forget what I'm trying to convey.
15	During my online study, I'm usually at ease when I'm writing in Chinese.
16	During my online study, I feel unsure of myself when I'm writing in Chinese.

Items 1-4, 5-8, 9-12, and 13-16 indicate factors of *Speaking*, *Listening*, *Reading*, and *Writing* anxiety respectively.

Appendix 3: Online Chinese Learning Strategies Scale (OCLAS)
Derived from Barnard-Brak et al. (2010)

(The questionnaire was presented in Thai, the native language of the participants.)

1	I set standards for my assignments in online courses.
2	I set short-term (daily or weekly) goals as well as long-term goals (monthly or for the semester).
3	I keep a high standard for my learning in my online courses.
4	I set goals to help me manage studying time for my online courses.
5	I don't compromise the quality of my work because it is online.
6	I choose the location where I study to avoid too much distraction.
7	I find a comfortable place to study.
8	I know where I can study most efficiently for online courses.
9	I choose a time with few distractions for studying for my online courses.
10	I try to take more thorough notes for my online courses because notes are even more important for learning online than in a regular classroom.
11	I read aloud instructional materials posted online to fight against distractions.
12	I prepare my questions before joining in the chat room and discussion.
13	I work extra problems in my online courses in addition to the assigned ones to master the course content.
14	I allocate extra studying time for my online courses because I know it is time-demanding.
15	I try to schedule the same time every day or every week to study for my online courses, and I observe the schedule.
16	Although we don't have to attend daily classes, I still try to distribute my studying time evenly across days.
17	I find someone who is knowledgeable in course content so that I can consult with him or her when I need help.
18	I share my problems with my classmates online so we know what we are struggling with and how to solve our problems.
19	If needed, I try to meet my classmates face-to-face.
20	I am persistent in getting help from the instructor through e-mail.
21	I summarize my learning in online courses to examine my understanding of what I have learned.
22	I ask myself a lot of questions about the course material when studying for an online course.
23	I communicate with my classmates to find out how I am doing in my online classes.
24	I communicate with my classmates to find out what I am learning that is different from what they are learning.

Items 1-5, 6-9, 10-13, 14-16, 17-20, and 21-24 indicate factors of *goal setting*, *environment structuring*, *task strategies*, *time management*, *help seeking*, and s*elf-evaluation* respectively.

Appendix 4: Words used for Chinese vocabulary size test

1. 安抚
2. 安宁
3. 安心
4. 败
5. 半
6. 抱怨
7. 遍地
8. 表格
9. 不
10. 厕所
11. 策划
12. 唱歌
13. 吵架
14. 诚实
15. 承诺
16. 愁
17. 出院
18. 处
19. 传说
20. 垂
21. 打包
22. 当地
23. 倒
24. 倒是
25. 地铁
26. 调查
27. 董事长
28. 发型
29. 粉丝
30. 份额
31. 服装
32. 干扰
33. 拐
34. 关怀
35. 广告
36. 规划
37. 好玩儿
38. 后果
39. 滑
40. 坏人
41. 辉煌
42. 回想
43. 活力
44. 活跃
45. 或许
46. 计算机
47. 焦点
48. 较量
49. 解决
50. 金融
51. 仅仅
52. 局
53. 巨头
54. 决赛
55. 开学
56. 肯定
57. 懒
58. 老实
59. 理
60. 绿色
61. 名牌儿
62. 模样
63. 内外
64. 念头
65. 培训班
66. 皮鞋
67. 平等
68. 普遍
69. 齐
70. 启事
71. 起飞
72. 器材
73. 倾销
74. 清洁工
75. 清醒
76. 求助
77. 曲
78. 驱动
79. 裙子
80. 人权
81. 人手
82. 认真
83. 伤员
84. 申请
85. 生日
86. 示威
87. 适宜
88. 收听
89. 四合院
90. 送
91. 拓展
92. 台
93. 体力
94. 条
95. 投票
96. 外文
97. 未
98. 无所谓
99. 下手
100. 先后
101. 先生
102. 消化
103. 协同
104. 新闻
105. 宣泄
106. 学校
107. 延
108. 一概而论
109. 一直
110. 遗产
111. 应对
112. 婴儿
113. 优
114. 友情
115. 有劲儿
116. 玉
117. 园
118. 原料
119. 月饼
120. 运转
121. 在乎
122. 挣扎
123. 整顿
124. 支柱
125. 注意
126. 注重
127. 自豪
